# The Tybee Leper

**Published:** 1903-05

**Authors:** 


### THE TYBEE LEPER.

THE newspapers have attempted to arouse alarm over the presence at Tybee Island of a leprous soldier. While it works a hardship on the railroad which has made a pleasure resort of Tybee and will doubtless cause some diminution in patronage of the resort by the summer visitor, there is assuredly no cause for fear. The popular dread of leprosy is an anachronism. It is a survival in the West of notions that anciently prevailed and still prevail to some extent in the East of the high communicability of the disease. Modern research has shown the fallacy of this conception and has abundantly demonstrated the fact of geographic and climatic immunity. With the exception of a few lepers in Louisiana and a handful of imported cases among Norwegian immigrants in the Northwest, the disease has never gained and can never gain a foothold in this country. Whatever be the nature and mode of contagion (and Jonathan Hutchinson is about the only one who still clings to the fish-host theory), the conditions in this country are absolutely inimical to its propagation. Leprosy, as a source of alarm on the score of extension, is infinitely less to be feared than tuberculosis. And yet the lethargy of the public toward the latter forms a violent contrast to its panicky terror of the former.

If the sentiment were reversed benefit of unconjecturable extent would be conferred upon mankind.

---

THE delay in the appearance of this and the April issue of THE JOURNAL-RECORD was due to a general strike among the union printers of Atlanta. Affairs seem now to be reasonably adjusted and no further delay in getting out THE JOURNAL is anticipated.

---

THE latest statistics show a marked diminution in the death-rate and in the number of emigrants from Ireland during the past year. All other statistics, however, show the great decrease which has been continuing for years in the population of Ireland. In 1824 there were over seven million inhabitants in Ireland, in 1845 over eight million, while in 1901 the population was a little under four million and a half. In 1902 the population was decreased by 17,756, showing a diminution in fifty-six years of almost four million people. Among the other statistics noted during the past year were the excess of births over deaths, 21,857; the loss by emigration, 39,613; the number of illegitimate births 2593, only two and one-half per cent. of the total for Great Britain. Out of a total of 79,119 deaths only 1263 were attributed to insanity.—*Philadelphia Medical Journal.*

---

The LaGrange Sanatorium was opened March 25th under the medical superintendency of Dr. Henry R. Slack, with Dr. F. M. Ridley, surgeon, and Dr. Henry W. Terrell, gynecologist. They have twelve beds, will give special attention to diseases of the stomach and intestines, and are prepared to do X-ray and electro-therapeutic work. We are pleased to learn that every room is now occupied.

